# S-Nitrosoglutathione Reductase Contributes to Thermotolerance by Modulating High Temperature-Induced Apoplastic H_2_O_2_ in *Solanum lycopersicum*

**DOI:** 10.3389/fpls.2022.862649

**Published:** 2022-04-12

**Authors:** Xuewei Song, Ting Wang, Yang Zhang, Jing-Quan Yu, Xiao-Jian Xia

**Affiliations:** ^1^Department of Horticulture, Zijingang Campus, Zhejiang University, Hangzhou, China; ^2^Key Laboratory of Horticultural Plants Growth, Development and Quality Improvement, Agricultural Ministry of China, Hangzhou, China

**Keywords:** GSNOR, nitric oxide, RBOH, reactive oxygen species, *S*-nitrosylation, thermotolerance

## Abstract

S-nitrosoglutathione reductase (GSNOR) is considered as a critical regulator of plant stress tolerance for its impacts on protein *S*-nitrosylation through regulation of the *S*-nitrosothiol (SNO) level. However, the mechanism of GSNOR-mediated stress tolerance is still obscure. Here, we found that GSNOR activity was induced by high temperature in tomato (*Solanum lycopersicum*) plants, whereas mRNA level of *SlGSNOR1* exhibited little response. Suppressing *SlGSNOR1* expression by virus-induced gene silencing (VIGS) increased accumulation of SNO and nitrites under high temperature and reduced thermotolerance. The compromised thermotolerance was associated with less accumulation of abscisic acid (ABA) and salicylic acid (SA), attenuated activation of mitogen-activated protein kinase (MAPK) and reduced expression of heat shock protein. Intriguingly, *SlGSNOR1* silencing impaired upregulation of *RESPIRATORY BURST OXIDASE HOMOLOG1* (*SlRBOH1*) and apoplastic H_2_O_2_ accumulation in response to high temperature, whereas *SlRBOH1* silencing abolished activation of GSNOR and led to a similar decline in thermotolerance as in *SlGSNOR1*-silenced plants. Importantly, H_2_O_2_ treatment recovered the thermotolerance and improved antioxidant capacity in *SlGSNOR1*-silenced plants. Our results suggest that GSNOR plays a role in regulating the *SlRBOH1*-dependent apoplastic H_2_O_2_ production in response to high temperature, while a balanced interaction between SNO and H_2_O_2_ is critical for maintaining the cellular redox homeostasis and thermotolerance.

## Introduction

Nitric oxide (NO), a reactive compound derived from cellular metabolism, acts as a ubiquitous signaling molecule in plants (Wilson et al., 2008). NO regulates seed germination, root architecture, nitrogen assimilation, stomatal movements, photosynthesis, and pathogen defense (Delledonne et al., 1998; Beligni and Lamattina, 2000; Desikan et al., 2002; Correa-Aragunde et al., 2004; Du et al., 2008; Wodala et al., 2008). NO is also a pivotal molecular regulator of abiotic stress response (Fancy et al., 2017). The large functional overlap between NO and hormones underpins substantial crosstalk between NO and hormonal signaling (Paris et al., 2013). NO acts downstream of NADPH oxidase-dependent H_2_O_2_ in mediating abscisic acid (ABA)-induced stomatal closure (Bright et al., 2006). However, it inhibits H_2_O_2_ synthesis and expression of wounding responsive genes downstream of jasmonic acid (JA) synthesis in tomato (Orozco-Cardenas and Ryan, 2002). NO is also a second messenger in auxin signal transduction during lateral and adventitious root formation (Correa-Aragunde et al., 2004). In addition, NO is involved in auxin biosynthesis, transport, and signaling for the maintenance of root stem cell niche in Arabidopsis (Sanz et al., 2014).

To be involved in signaling pathways, the production of NO needs to be under strict control. Until now, several potential routes of NO production have been unraveled in plants. Although in animals nitric oxide synthase (NOS)-mediated conversion from L-arginine to L-citrulline is the primary pathway for NO release, the counterparts in plants with similar biochemical characteristics as NOS have not yet been identified (Guo et al., 2003; Moreau et al., 2008). Importantly, nitrate reductase (NR) is the major player of NO production in plants (Bright et al., 2006; Wilson et al., 2008). In addition, NO can be synthesized in chloroplast, mitochondria, and peroxisome (Planchet et al., 2005; Jasid et al., 2006; Corpas et al., 2009).

In contrast to the controversy regarding the NO production pathways, the metabolism of NO is relatively clear. NO can react with reactive oxygen species (ROS), forming peroxynitrite, or can be scavenged by hemoglobin (Delledonne et al., 2001; Perazzolli et al., 2004). Furthermore, glutathione (GSH) which exists in large abundance in cells can react with NO to form *S*-nitrosoglutathione (GSNO), which can then be metabolized by the enzyme GSNO reductase (GSNOR; Astier et al., 2011). Interestingly, GSNO is a reservoir of NO in plant cells and can mediate protein *S*-nitrosylation (Wang et al., 2006). It is clear that dynamic *S*-nitrosylation is a regulatory mechanism comparable to phosphorylation in signal transduction (Perazzolli et al., 2004; Belenghi et al., 2007; Paris et al., 2013). In recent past, several targets of protein *S*-nitrosylation with confirmed physiological functions have been identified (Lindermayr et al., 2006; Romero-Puertas et al., 2007; Tada et al., 2008; Palmieri et al., 2010; Yun et al., 2011, 2016; Terrile et al., 2012; Wang et al., 2015; Yang et al., 2015). The identified *S*-nitrosylated proteins are involved in hormone signaling, defense, antioxidant, and metabolism. Recent studies found that *S*-nitrosation is a ubiquitous NO-dependent signaling mechanism (Astier et al., 2019), which means NO is not only a signaling molecule, but also as a free radical determines the posttranslational modifications through cysteine *S*-nitrosylation and tyrosine nitration (León and Costa-Broseta, 2020). In addition, NO bioactivity regulates a lot of post-translational modifications, such as SUMOylation, phosphorylation and acetylation (Mengel et al., 2017; Del Castello et al., 2019; Skelly et al., 2019; Gupta et al., 2020). As the research in NO signaling advances, the list of *S*-nitrosylation targets and related physiological functions are still increasing.

It is to be noted that denitrosylation, one another facet of signaling is less understood. Recently, the roles of GSNOR that modulates protein *S*-nitrosylation by regulating the spatial-temporal availability of GSNO, in plant development and stress response are clear (Malik et al., 2011). Loss of *GSNOR1* function in Arabidopsis results in higher level of GSNO and reduced basal and *R*-gene- mediated resistance (Feechan et al., 2005). The compromised resistance is associated with downregulation of defense genes (Xu et al., 2013). However, reducing GSNOR1 activity in transgenic antisense lines enhanced basal resistance against oomycete (Rusterucci et al., 2007). The seemingly contrasting role of GSNOR in plant defense may be caused by different level of *S*-nitrosylated proteins such as NPR1 (Malik et al., 2011). Apart from defense against pathogens, GSNOR also seems to play roles in abiotic stress response. GSNOR activity has been shown to be induced by chilling, high temperature and wounding in different crop species (Corpas et al., 2008; Airaki et al., 2012; Kubienova et al., 2014). Of particular interest, Arabidopsis *GSNOR1* loss-of-function mutant failed to acclimate to high temperature (Lee et al., 2008). However, high temperature inhibited GSNOR activity in hypocotyl of sunflower seedlings (Chaki et al., 2011), suggesting that responses of GSNOR to high temperature may differ according to plant species and/or tissues.

Despite an established role of GSNOR in thermotolerance in model plants, the critical roles of GSNOR in thermotolerance as well as the underlying mechanism of GSNOR-mediated thermotolerance in economically important crops still remain elusive. In this study, we used virus-induced gene silencing (VIGS) to suppress the expression of *GSNOR* in tomato plants and studied its role in thermotolerance. In addition, we examined activity of mitogen-activated protein kinase (MAPK), stress hormone levels and ROS metabolism during heat stress. This study unveiled the role of apoplastic H_2_O_2_ in GSNOR-mediated thermotolerance in tomato.

## Materials and Methods

### Plant Materials, Virus-Induced Gene Silencing, and Growth Conditions

The germinated tomato (*Solanum lycopersicum* L. cv. Ailsa Craig) seeds were grown in a mixed medium comprised of peat and vermiculite (2:1; v/v) in an incubation room under the condition of 12 h light (PPFD, 200 μmol m^–2^ s^–1^) at 21°C and 12 h dark at 16°C. When the cotyledons fully expanded, VIGS was performed as described previously (Xia et al., 2014). Briefly, fragments of genes encoding *S*-nitrosoglutathione reductase1 (*SlGSNOR1*, SGN accession Solyc09g064370.2.1) and RESPIRATORY BURST OXIDASE HOMOLOG 1 (*SlRBOH1*, SGN accession Solyc08g081690.2.1) were amplified using gene-specific primers: *SlGSNOR1*, 5′-tgctctagaAGCAACCCATTCAGCAAGTC-3′ and 5′-cgc ggatccTGTTTATGTCCGCAAGTGTC-3′ (with *Xba*I and *Bam*HI restriction sites); *SlRBOH1*, 5′-atacggagctcAAGAA TGGGGTTGATATTGT-3′ and 5′-ataccgctcgagCTCTGACTTAT TCCTTAC-3′ (with *Sac*I and *Xho*I restriction sites). The fragments were cloned into the pTRV2 vector. Empty pTRV2 vector was used as a control. All constructs were confirmed by sequencing and subsequently transformed into the *Agrobacterium tumefaciens* strain GV3101. A mixture of *A. tumefaciens* carrying pTRV1 and pTRV2 derivative (1:1, v/v, OD_600_ = 0.6) was infiltrated into the fully expanded cotyledons. pTRV1 encodes the replication and movement viral functions that help gene silencing through pTRV2. Seedlings were then covered with plastic films and kept in dark for 3 days. When the second true leaves fully expanded, the seedlings were transplanted into pots (15 cm diameter and 15 cm height).

For evaluating the thermotolerance, plants at five-leaf stage were transferred into growth chambers. Plants were first kept in control condition for 2 days for acclimation. Thereafter, plants were exposed to high temperature 42°C/38°C (day/night). The photoperiod and PPFD were the same as control growth condition. The plants used for control were maintained in growth chambers at 21°C/16°C. Stress tolerance was measured based on changes in the maximal quantum yield (Fv/Fm), electrolyte leakage and malondialdehyde (MDA) content. To study the effects of H_2_O_2_ on thermotolerance, 5 mM H_2_O_2_ solution was sprayed onto the leaves.

### Analysis of Chlorophyll Fluorescence, Electrolyte Leakage, and Malondialdehyde Contents

Chlorophyll fluorescence was measured using an Imaging-PAM Chlorophyll Fluorometer (IMAG-MAXI, Heinz Walz, Effeltrich, Germany). Before measurements, the plants were dark-adapted for 30 min. The initial fluorescence (Fo) was obtained after switching on the measuring beam, and then the maximum fluorescence (Fm) was obtained after applying a 0.8s saturating pulse (>4,000 μmol m^–2^ s^–1^). Fv/Fm was calculated as (Fm-Fo)/Fm and was determined using whole leaflet as area of interest.

Lipid peroxidation was determined by quantifying the MDA equivalents using 2-thiobarbituric acid (TBA) as described by Hodges et al. (1999). Electrolyte leakage in leaves was determined according to the method described by Bajji et al. (2002).

### Determination of Total *S*-Nitrosothiols and Nitrites Content

Total *S*-Nitrosothiols (SNO) content was measured using the Saville-Griess assay as described by Lin et al. (2012). Briefly, plant tissues were powdered in liquid nitrogen and lysed with 600 μL of extraction buffer (50 mM Tris-HCl, pH 8.0, and 150 mM NaCl) containing 1 mM phenylmethanesulfonyl fluoride (PMSF) and incubated on ice for 20 min. After incubation, samples were centrifuged at 10,000 g for 15 min at 4°C. Then, 160 μL of supernatant was incubated with the same volume of 1% sulfanilamide and 0.1% *N*-(1-naphthyl)-ethylenediamine with or without the addition of 3.75 mM HgCl_2_ for 20 min in the dark. Then, SNO content was measured spectrophotometrically at 540 nm and calculated by using GSNO concentration standard curve.

To determine the accumulation of nitrites, 0.3 g tomato leaves were homogenized using 50 mM glacial acetic acid (pH 3.6) in ice bath and centrifuged at 12,000 g for 15 min. Aliquot supernatant was mixed with Griess reagent (Sigma-Aldrich, United States) and reacted for 30 min in 25°C. The content of nitrites was calculated based on the absorbance of the reaction mixture at 540 nm.

### H_2_O_2_ Quantification, Histochemical Staining, and Subcellular Localization

For determination of H_2_O_2_ content in leaves, 0.3 g samples were homogenized in 3 mL of pre-cooled HClO_4_ (1.0 M) using pre-chilled mortar and pestle. The content of H_2_O_2_ in the extracts was determined according to the method described by Willekens et al. (1997).

Histochemical staining of H_2_O_2_ was performed as described previously (Thordal-Christensen et al., 1997) with minor modifications. Leaflets were vacuum infiltrated with 1 mg mL^–1^ 3,3’-diaminobenzidine (DAB) in 50 mM TRIS-acetate buffer (pH 3.8) and incubated at 25°C in the dark for 6 h. Samples were rinsed in 80% (v/v) ethanol for 10 min at 70°C and mounted in lactic acid/phenol/water (1:1:1, v/v/v). H_2_O_2_ accumulation was detected by an Olympus motorized system microscope (BX61, Olympus, Tokyo, Japan).

Subcellular localization of H_2_O_2_ was visualized using CeCl_3_. Sections were prepared as described previously (Zhou et al., 2014). As the negative control, leaf segments were incubated with 1 mM ascorbate for 2 h (Supplementary Figure 1). Electron-dense CeCl_3_ deposits which are formed in the presence of H_2_O_2_ are examined by a transmission electron microscope (JEOL TEM-1230EX) at an accelerating voltage of 75 kV.

### Determination of Salicylic Acid and Abscisic Acid

Determination of Salicylic Acid (SA) was performed using a biosensor method according to DeFraia et al. (2008). Leaf tissue of 0.1 g was grounded into powder with liquid nitrogen, and then 200 μL of acetate buffer (0.1 M, pH 5.6) was added. Samples were then mixed thoroughly and centrifuged for 15 min at 16,000 g. Half of the supernatant was stored on ice for free SA measurement and half was incubated at 37°C for 90 min with 4 U of β-glucosidase (Sigma-Aldrich, St. Louis, MO, United States) for conjugated SA measurement. Culture of *Acinetobacter* sp. ADPWH_*lux* was diluted in 37°C LB (1:20) and grown for 3 h at 200 rpm to an OD_600_ of 0.4. Leaf extract (20 μL), LB (60 μL), and biosensor culture (50 μL) were added to the wells of a black 96-well black plate. The plate was incubated at 37°C for 1 h without shaking and then the luminescence was read with a Perkin Ellmer EnSpire Multilabel Plate Reader (PerkinElmer, Waltham, MA, United States).

For ABA measurement, sample of 1 g was homogenized in extraction solution (80% methanol, v/v). The extracts were centrifuged at 10,000 g for 20 min. The supernatant was eluted through a Sep-Pak C18 cartridge (Waters, Milford, MA, United States) to remove the polar compounds and subsequently stored at -20°C for an ELISA. ELISA was performed following the manufacturer’s instructions (China Agricultural University, Beijing, China). ABA was determined using a Perkin Ellmer EnSpire Multilabel Plate Reader (PerkinElmer, Waltham, MA, United States).

### Mitogen-Activated Protein Kinase Assay

Samples were ground to fine powder in liquid nitrogen and solubilized in extraction buffer (100 mM HEPES, pH 7.5, 5 mM EDTA, 5 mM EGTA, 10 mM DTT, 10 mM Na_3_VO_4_, 10 mM NaF, 50 mM β-glycerophosphate, 1 mM phenylmethylsulfonyl fluoride, 5 μg mL^–1^ antipain, 5 μg mL^–1^ aprotinin, 5 μg mL^–1^ leupeptin, 10% glycerol, and 7.5% polyvinylpolypyrrolidone). The extracts were centrifuged at 12,000 g for 20 min. Denatured protein extracts were then separated by SDS-PAGE and blotted onto PVDF membrane (Biorad). Immunoblots were blocked in 5% (w/v) BSA (Sigma) in TBS-Tween (0.1%) for 1–2 h. The activated MAP kinases were detected using anti-phospho-p44/42 MAPK (Erk1/2) primary antibody according to our previous study (Wang et al., 2019) (1:1000, Cell Signaling Technology) overnight, followed by anti-rabbit-HRP conjugated secondary antibodies (Cell Signaling Technology).

### Assays of *S*-Nitrosoglutathione Reductase and Antioxidant Enzymes

The activity of *S*-nitrosoglutathione reductase (GSNOR) was measured as described previously (Sakamoto et al., 2002). GSNOR was extracted using buffer (50 mM HEPES, pH 8.0; 20% glycerol; 10 mM MgCl_2_; 1 mM EDTA; 1 mM EGTA; 1 mM benzamidine; and 1 mM ε-aminocaproic acid). The homogenate was centrifuged at 4°C, 16,000 g for 15 min and the supernatant was desalted using spin columns (Thermo Fisher Scientific, Rockford, IL, United States). The reaction system was as follows: 30 μL protein samples, 300 μL reaction buffer (20 mM Tris-HCl, pH 8.0, 0.2 mM NADH, 0.5 mM EDTA), and 400 μM GSNO. The activity of GSNOR was determined by monitoring the decomposition of NADH.

For extraction of antioxidant enzymes, 0.3 g samples were homogenized in 50 mM potassium phosphate buffer (pH 7.0) containing 0.1 mM EDTA and 1% polyvinylpyrrolidone (w/v). The homogenate was centrifuged at 12,000 g for 15 min at 4°C. The supernatant was used to measure the activities of superoxide dismutase (SOD), ascorbate peroxidase (APX), catalase (CAT), and glutathione reductase (GR). SOD activity was assayed by its ability to inhibit the photochemical reduction of NBT as described by Ahammed et al. (2013). The APX activity was determined by the method of Nakano and Asada (1981). The CAT activity was measured by the method as described by Cakmak and Marschner (1992). The GR activity were measured and calculated as previously described (Foyer and Halliwell, 1976).

### Total RNA Extraction and Gene Expression Analysis

RNA was extracted using RNAprep pure Plant Kit (TIANGEN, Beijing, China) according to the operation manual. Total RNA (0.5 mg) was reverse transcribed to cDNA using ReverTra Ace qPCR RT Kit with genome-DNA-removing enzyme (Toyobo, Osaka, Japan). Quantitative real-time PCR was performed using the iCycler iQ real-time PCR detection system (Bio-Rad, Hercules, CA, United States). The reaction system (20 mL) was as follows: 10 mL SYBR (Takara, Japan), 0.2μL sense and antisense primer, 1 mL cDNA and 8.6 mL ddH_2_O. The PCR conditions consisted of denaturation at 95°C for 3 min, followed by 40 cycles of denaturation at 95°C for 30 s, annealing at 58°C for 30 s, and extension at 72°C for 30 s. Relative transcript level was calculated according to the method of Livak and Schmittgen (2001). *Actin* was used as a reference gene. Primers for qRT-PCR were listed in Supplementary Table 1.

### Statistical Analysis

The experimental design was a completely randomized design. Data were subjected to statistical analysis of variance (ANOVA) using the SAS package (SAS9.4). The differences between the means were separated by Tukey’s test at a level of *P* < 0.05 (Supplementary Table 2).

## Results

### Silencing of *SlGSNOR1* Disrupted *S*-Nitrosothiol Metabolism

**FIGURE 1:**
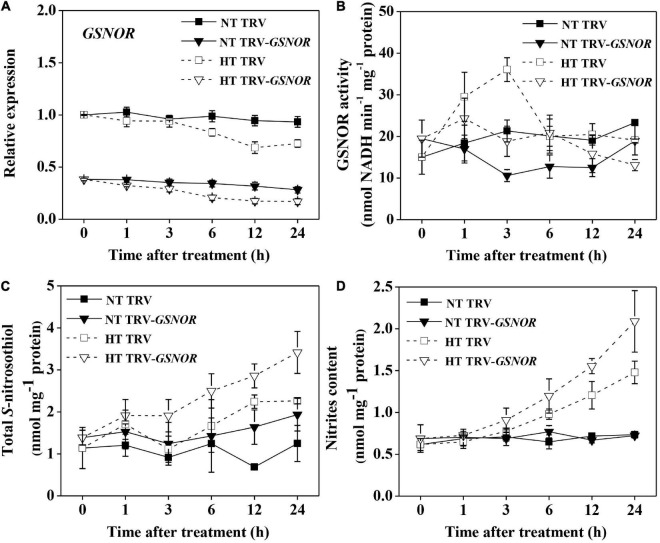
Relative transcript level of *SlGSNOR1* **(A)**, enzymatic activity of GSNOR **(B)**, total *S*-nitrosothiol (SNO) content **(C)**, and nitrites content **(D)** in *SlGSNOR1*-silenced or TRV-empty vector infiltrated plants in response to heat stress. Plants in the five-leaf stage were exposed to high temperature (42°C/38°C) or normal temperature (21°C/16°C). Data are means of five replicates (±SD). NT, normal temperature; HT, high temperature.

To study the mechanism by which SNO metabolism regulates thermotolerance, we silenced *SlGSNOR1* using VIGS in tomato plants and first studied the SNO metabolism in response to high temperature. Gene expression analysis confirmed that the expression of *SlGSNOR1* was suppressed by *c.a.* 70% following VIGS (Figure 1A). The expression of *SlGSNOR1* was not significantly affected by high temperature during initial phase (<3 h) of heat treatment, but was slightly downregulated at later time in both silenced and TRV plants (TRV-empty vector infiltrated). The basal activity of GSNOR at normal temperature was generally reduced by gene silencing during the experiment (Figure 1B). In contrast to the transcriptional changes, activity of GSNOR was significantly induced by high temperature during the initial phase (<3 h), and then declined rapidly. However, the temporary increase in GSNOR activity was inhibited by silencing. Silencing of *SlGSNOR1* did not significantly affect total SNO content at normal temperature (Figure 1C), whereas it led to higher accumulation of total SNO at high temperature. By contrast, total SNO content was less affected by high temperature in TRV plants. The changes in nitrites content followed the same trend as of total SNO except for the nitrites content in TRV plants under high temperature which was evident by a significant increase (Figure 1C). These results indicate the importance of *SlGSNOR1* in SNO metabolism at high temperature.

### Silencing of *SlGSNOR1* Inhibited Thermotolerance

**FIGURE 2:**
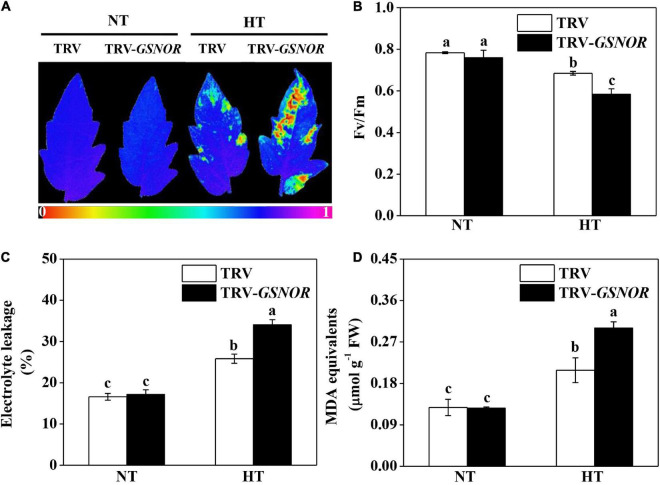
Silencing of *SlGSNOR1* compromised thermotolerance. **(A)** Images of maximum quantum yield (Fv/Fm) of leaves after 24 h exposure to high temperature (42°C/38°C) or normal temperature (21°C/16°C). The pseudocolor scale at the bottom of the image ranges from 0 (black) to 1 (purple). **(B–D)** Fv/Fm values, electrolyte leakage and malondialdehyde (MDA) content in leaves after 24 h exposure to high temperature. Data are means of five replicates (±SD). Means denoted by the same letter did not significantly differ at *P* < 0.05, according to Tukey’s test. NT, normal temperature; HT, high temperature.

The maximum quantum yield (Fv/Fm), which indicates the functional integrity of photosystem II (PSII), was significantly reduced by high temperature (Figures 2A,B). Meanwhile, high temperature stress caused significant increase in electrolyte leakage and malondialdehyde (MDA) content (Figures 2C,D), both as indicators of membrane damage. Importantly, *SlGSNOR1* silencing exacerbated the inhibition of Fv/Fm and increase of electrolyte leakage and MDA content. The results confirmed the role of GSNOR in thermotolerance in tomato.

**FIGURE 3:**
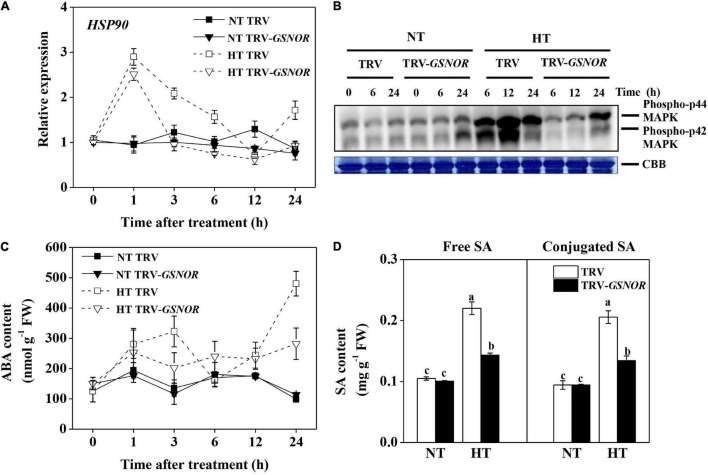
Relative transcript level of *HSP90* **(A)**, activation of MAP kinase **(B)**, accumulation of ABA **(C)**, and accumulation of free and glucose-conjugated SA **(D)** in *SlGSNOR1*-silenced or TRV-empty vector infiltrated plants in response to high temperature (42°C/38°C) or normal temperature (21°C/16°C). For SA measurement, samples were taken at 24 h after heat stress. Data are means of five replicates (± SD). Means denoted by the same letter did not significantly differ at *P* < 0.05, according to Tukey’s test. NT, normal temperature; HT, high temperature.

Heat shock proteins (HSPs) are critical for thermotolerance. *HSP90* was rapidly induced by high temperature at 1 h (Figure 3A). Then, the expression gradually declined to the control level at 12 h, and was induced again at 24 h. By contrast, *SlGSNOR1* silencing compromised the biphasic induction of *HSP90*. Although the first peak of *HSP90* induction was less affected, the expression at later time points was inhibited and the second peak was completely blocked.

MAPK are upstream regulators of stress response. MAPK was activated by high temperature (Figure 3B). Intriguingly, the activation of MAPK was delayed and attenuated by *SlGSNOR1* silencing. Only a moderate activation of MAPK was observed at 24 h after high temperature stress.

ABA and SA are stress hormones, which play critical roles in thermotolerance. Consistent with the expression of *HSP90*, ABA showed a biphasic change in response to high temperature, showing accumulation peaks at 3 and 24 h (Figure 3C). Importantly, silencing of *SlGSNOR1* strongly inhibited accumulation of ABA during high temperature stress. Similarly, accumulations of free and conjugated SA at 24 h after high temperature stress were inhibited by *SlGSNOR1* silencing (Figure 3D).

### Silencing of *SlGSNOR1* Impaired Apoplastic H_2_O_2_ Accumulation and Antioxidant Metabolism at High Temperature

**FIGURE 4:**
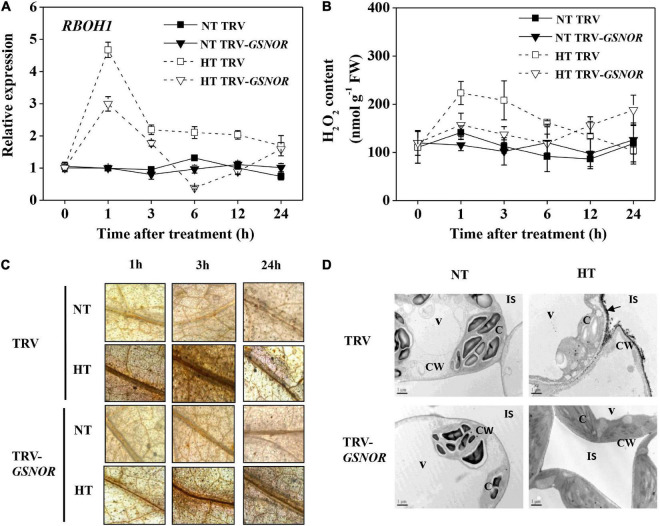
Silencing of *SlGSNOR1* impaired apoplastic H_2_O_2_ accumulation in response to high temperature. **(A)** Relative transcript level of *SlRBOH1*, encoding NADPH oxidase. **(B)** Chemical quantification of H_2_O_2_ in leaves. **(C)** Histochemical detection of H_2_O_2_ in leaves using 3, 3’-diaminobenzidine (DAB). **(D)** Subcellular localization of H_2_O_2_ leaf cells using CeCl_3_. Black arrows indicate apoplastic H_2_O_2_ accumulation. Samples were taken at 3 h after heat stress. C, chloroplast; CW, cell wall; IS, intercellular space; V, vacuole. Data are means of five replicates (±SD). NT, normal temperature; HT, high temperature.

To further study the mechanism of how GSNOR regulates thermotolerance, we analyzed the production and metabolism of ROS, which should be strictly controlled in response to stress. *SlRBOH1*, encoding NADPH oxidase which is responsible for apoplatic H_2_O_2_ accumulation, was rapidly upregulated by high temperature at 1 h (Figure 4A). Thereafter, its expression declined and was maintained at a moderately high level. However, silencing of *SlGSNOR1* compromised the upregulation of *SlRBOH1* during high temperature stress. Consistent with the expression of *SlRBOH1*, H_2_O_2_ content was significantly increased within the first 3 h following high temperature stress (Figure 4B). Then H_2_O_2_ content declined to a basal level. Interestingly, *SlGSNOR1* silencing inhibited the initial increase of H_2_O_2_ accumulation; however, significantly increased H_2_O_2_ content 24 h after stress. Next, we detected ROS accumulation at tissue and cellular level by DAB and CeCl_3_ staining, respectively. High temperature induced a strong accumulation of H_2_O_2_ in vascular tissue (Figure 4C), especially in the initial phase (<3 h). Further analysis indicated that high temperature-induced H_2_O_2_ was mainly localized in apoplast (Figure 4D). However, the initial induction of H_2_O_2_ in the vascular tissue and apoplast following high temperature stress was strongly inhibited by *SlGSNOR1* silencing.

**FIGURE 5:**
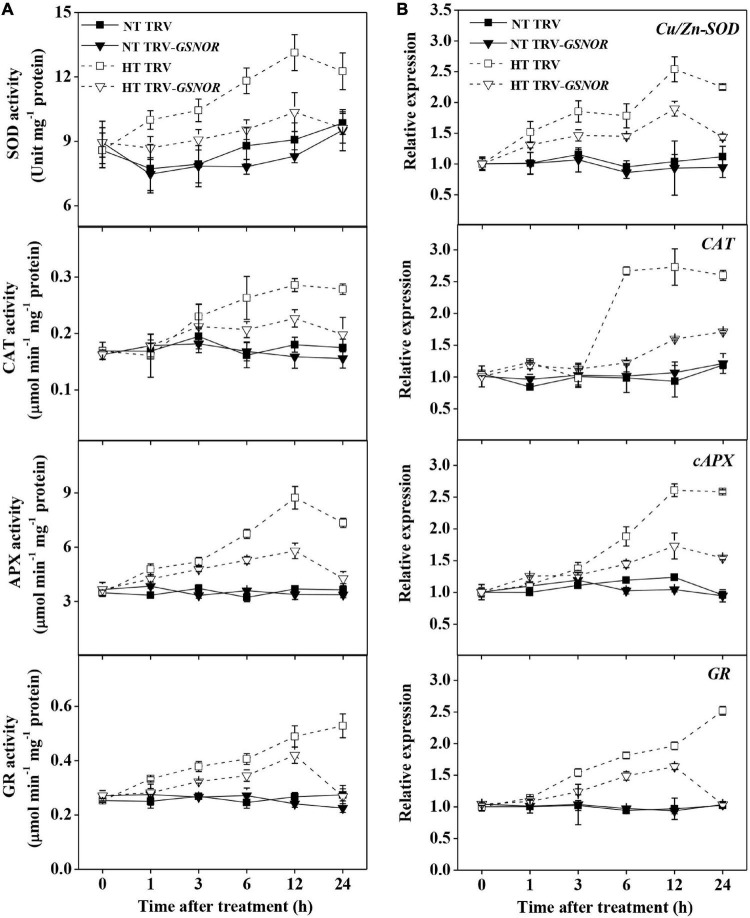
Silencing of *SlGSNOR1* compromised upregulation of antioxidant capacity in response to high temperature. **(A)** Changes in the activity of antioxidant enzymes, superoxide dismutase (SOD), catalase (CAT), ascorbate peroxidase (APX), and glutathione reductase (GR). **(B)** Changes in the relative transcript levels of antioxidant genes, *Cu/Zn-SOD*, *cAPX*, *CAT* and *GR*. Data are means of three to five replicates (± SD). NT, normal temperature; HT, high temperature.

ROS trigger the upregulation of antioxidant system. Along with the H_2_O_2_ production, the activity of antioxidant enzymes, superoxide dismutase (SOD), catalase (CAT), ascorbate peroxidase (APX), and glutathione reductase (GR), were significantly increased by high temperature stress (Figure 5A). The maximum induction of these enzymes occurred at 12–24 h after high temperature stress, which was preceded by the initial increase of H_2_O_2_ content. In analogy to the changes of enzyme activity, the expression of antioxidant genes, *Cu/Zn-SOD*, *CAT*, *cAPX* and *GR*, were also upregulated (Figure 5B). However, *SlGSNOR1* silencing strongly inhibited the induction of both activity and gene expression of the antioxidant enzymes, especially from 12 to24 h during high temperature stress.

### Silencing of *SlRBOH1* Impaired *S*-Nitrosothiol Metabolism and Thermotolerance

**FIGURE 6:**
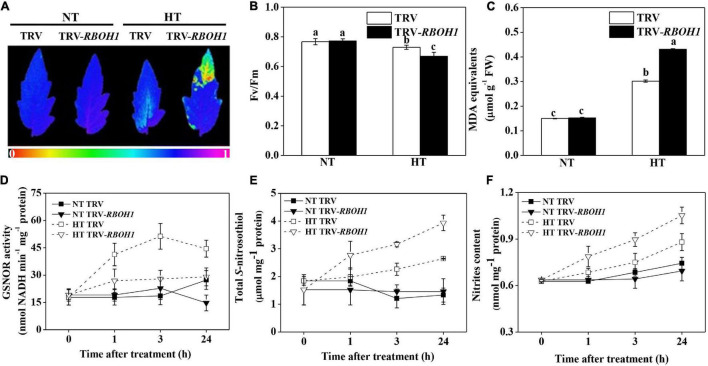
Silencing of *SlRBOH1* compromised thermotolerance and impaired SNO metabolism. **(A)** Images of the maximum quantum yield (Fv/Fm) of leaves after exposure to high temperature (42°C/38°C) or normal temperature (21°C/16°C). The pseudocolor scale at the bottom of the image ranges from 0 (black) to 1 (purple). **(B,C)** Fv/Fm values and malondialdehyde (MDA) content in leaves after exposure to heat stress; **(D–F)** Enzymatic activity of GSNOR, total *S*-nitrosothiol (SNO) content and nitrites content after heat stress. Data are means of five replicates (±SD). Means denoted by the same letter did not significantly differ at *P* < 0.05, according to Tukey’s test. NT, normal temperature; HT, high temperature.

The above results provided an assumption that the compromised thermotolerance in *SlGSNOR1*-silenced plants might be related to failure of rapid induction of *SlRBOH1* expression and H_2_O_2_ accumulation in response to high temperature. Next, we analyzed the response of *SlRBOH1*-silenced plant to high temperature. Silencing of *SlRBOH1* led to stronger decline in Fv/Fm and higher accumulation of MDA as compared to TRV plants following high temperature stress (Figures 6A–C). Interestingly, compromised thermotolerance of *SlRBOH1*-silenced plants was associated with disrupted SNO metabolism. Induction of GSNOR activity by high temperature was significantly compromised by *SlRBOH1* silencing, whereas GSNOR activity at normal temperature was not affected (Figure 6D). Accordingly, silencing of *SlRBOH1* led to higher accumulation of total SNO and nitrites at high temperature (Figures 6E,F). The results confirmed the involvement of NADPH oxidase in thermotolerance and supported the notion that GSNOR contributes to thermotolerance by regulating NADPH oxidase-dependent ROS.

### H_2_O_2_ Treatment Recovered the Thermotolerance of *SlGSNOR1*-Silenced Plants

**FIGURE 7:**
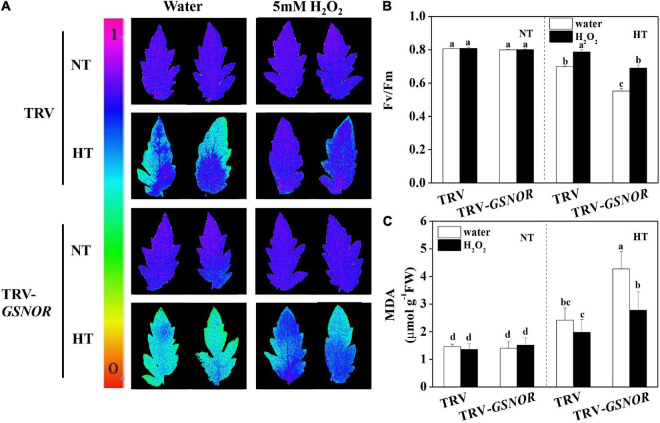
H_2_O_2_ recovered the thermotolerance in *SlGSNOR1*-silenced plants. **(A)** Images of the maximum quantum yield (Fv/Fm) of leaves after 24 h exposure to high temperature (42°C/38°C) or normal temperature (21°C/16°C). The pseudocolor scale at the left side of the image ranges from 0 (black) to 1 (purple); **(B,C)** Fv/Fm values and malondialdehyde (MDA) content in leaves after 24 h exposure to heat stress. Data are means of five replicates (±SD). Means denoted by the same letter did not significantly differ at *P* < 0.05, according to Tukey’s test. NT, normal temperature; HT, high temperature.

To further study the role of ROS in *SlGSNOR1*-mediated thermotolerance, we analyzed the effects of H_2_O_2_ treatment on thermotolerance in *SlGSNOR1*-silenced plants. Interestingly, Fv/Fm and MDA content at high temperature were dramatically increased and decreased, respectively by H_2_O_2_ treatment in *SlGSNOR1*-silenced plants. In addition, H_2_O_2_ treatment slightly increased Fv/Fm and decreased MDA content in TRV plants following high temperature stress (Figures 7A,B).

**FIGURE 8:**
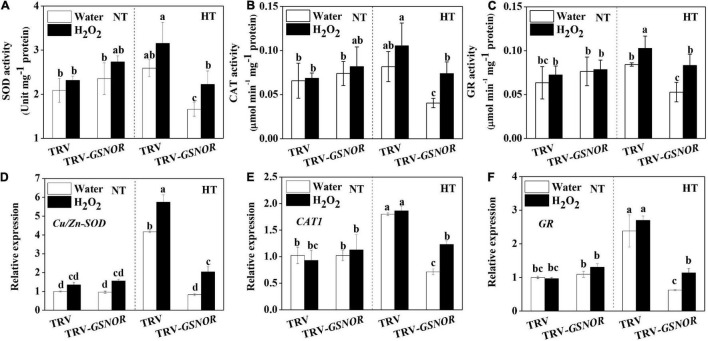
H_2_O_2_ recovered the upregulation of antioxidant capacity in response to high temperature in *SlGSNOR1*-silenced plants. **(A–C)** Changes in the activity of antioxidant enzymes, superoxide dismutase (SOD), catalase (CAT), and glutathione reductase (GR). **(D–F)** Changes in the relative transcript levels of antioxidant genes, *Cu/Zn-SOD*, *CAT*, and *GR*. Plants in the five-leaf stage were exposed 24 h to high temperature (42°C/38°C) or normal temperature (21°C/16°C). Data are means of three to five replicates (±SD). NT, normal temperature; HT, high temperature.

The improved thermotolerance in *SlGSNOR1*-silenced plants after H_2_O_2_ treatment was accompanied by increase in antioxidant capacity. In TRV plants, activity of SOD, CAT, and GR was induced by high temperature, whereas the induction was enhanced by H_2_O_2_ treatment (Figures 8A–C). By contrast, high temperature failed to induce the activity of antioxidant enzymes in *SlGSNOR1*-silenced plants. Interestingly, H_2_O_2_ significantly increased the activity of antioxidant enzymes at high temperature in *SlGSNOR1*-silenced plants. Similarly, expression of corresponding antioxidant genes was significantly enhanced by H_2_O_2_ at high temperature (Figures 8D–F). Taken together, the results indicated that H_2_O_2_ treatment recovered the thermotolerance in *SlGSNOR1*-silenced plants.

## Discussion

A balanced production and turnover of GSNO is critical for the overall status of protein *S*-nitrosylation, which affects plant growth and stress response (Wang et al., 2006; Astier et al., 2011). High temperature has been shown to induce accumulation of NO and SNO in different plant species, leading to nitrosative stress as indicated by tyrosine-nitrated proteins including those involved in CO_2_ assimilation (Chaki et al., 2011; Airaki et al., 2012; Leterrier et al., 2012; Cheng et al., 2018). In addition, *in vivo* evidence shows that PSII photochemical reaction is inhibited by GSNO (Wodala et al., 2008). Here, our experiment using intact plants showed that inhibition of GSNOR by VIGS led to excessive accumulation of SNO and nitrites, which was associated with photoinhibition as shown by decline in Fv/Fm at high temperature (Figures 1, 2). This is consistent with previous study using detached leaf disks in Arabidopsis and confirms the roles of GSNOR in protecting photosynthetic apparatus at high temperature (Lee et al., 2008; Chaki et al., 2011). In contrast to the thermotolerance, suppressing GSNOR activity by VIGS in tomato did not cause aberrant development as observed in GSNOR loss-of-function mutant of Arabidopsis (Lee et al., 2008). Silencing of *SlGSNOR1* in tomato did not completely eliminate the expression of *GSNOR*, thus the basal activity may be sufficient for growth and development in normal conditions.

Regarding the regulation of GSNOR, different results have been reported, showing that GSNOR protein/activity was either not affected or inhibited by high temperature (Corpas et al., 2008; Chaki et al., 2011; Airaki et al., 2012; Kubienova et al., 2014). In our study, transcript of *SlGSNOR1* was not significantly affected and even was slightly inhibited by high temperature. However, GSNOR activity was induced by high temperature during early hours of stress imposition (Figure 1). The discrepancy regarding GSNOR activity may be due to different sampling time after stress. Alternatively, the regulation of GSNOR activity by high temperature may be species specific. Our results combined with previous study suggested that GSNOR may not be regulated at transcriptional or translational level, but most probably at posttranslational level. Interestingly, induction of GSNOR activity by high temperature was abolished in *SlRBOH1*-silenced plants (Figure 6), indicating that NADPH oxidase-dependent ROS play a role in regulating the GSNOR activity. Recent studies found that calmodulins and redox signal are involved in the regulation of GSNOR activity (Zhang et al., 2020; Chae et al., 2021; Li et al., 2021). It was found that NADPH-dependent glutaredoxin (GRX) and thioredoxin (TRX) as transmitters of ROS signal regulate the function of proteins through thiol redox exchanges (Meyer et al., 2012). In addition, calcium signaling is closely associated with RBOH-mediated ROS signaling (Xia et al., 2015). Taken together, these results suggested that GSNOR activity may be posttranslationally regulated by RBOH mediated calcium and redox signals.

Apart from protection against nitrosative stress, GSNOR plays a role in signaling processes in response to high temperature. *S*-nitrosylation-mediated NO signaling converges with plant hormone networks through regulating hormonal biosynthesis and signaling (Paris et al., 2013). Loss-of-function of *GSNOR* leads to impaired biosynthesis of SA in Arabidopsis when plants are challenged with pathogens (Feechan et al., 2005). In this study, silencing of *SlGSNOR1* compromised the biosynthesis of ABA and SA in response to high temperature (Figure 3). ABA is the major player mediating plant adaptation to diverse abiotic stresses (Roychoudhury et al., 2013). Emerging evidence also supports the role of SA in thermotolerance (Larkindale et al., 2005; Clarke et al., 2009). Attenuated increase of ABA and SA accumulation was associated with compromised upregulation of antioxidant system, which is critical for preventing oxidative damages (Figure 5). Although the mechanism of decreased accumulation of ABA and SA in *SlGSNOR1*-silenced plants at high temperature is not clear, the effects of SNO on hormone biosynthesis are thought to be mediated at the transcriptional level (Malik et al., 2011). Interestingly, activation of MAPK, the upstream regulator of stress response, was inhibited by *SlGSNOR1* silencing at high temperature (Figure 3). When plants are exposed to environmental stimuli, transcription factors controlling the expression of early response genes are targets of MAPK-mediated phosphorylation (Andreasson and Ellis, 2010), whereas MAPK itself is a target of redox regulation (Matern et al., 2015). It appears that GSNOR-mediated redox homeostasis contributes to regulation of MAPK, which in turn plays roles in gene expression and stress response.

A striking feature of high temperature response in *SlGSNOR1*-silenced plants is the inhibition of apoplastic H_2_O_2_ accumulation (Figure 4). The wide source of ROS production and feedback mechanism involving interplay between hormones and ROS make it difficult to interpret the primary target sites responsible for the decreased ROS production (Xia et al., 2015). From the apoplastic localization and expression of *SlRBOH1* gene, we hypothesized that NADPH oxidase may be inhibited due to *SlGSNOR1* silencing. NADPH oxidase-dependent ROS are second messengers mediating signaling processes, such as MAPK activation, hormone biosynthesis and gene expression in response to a wide range of stresses (Mittler et al., 2011; Baxter et al., 2014). Indeed, *SlRBOH1* silencing led to similar inhibition in thermotolerance as observed by silencing of *SlGSNOR1* (Figure 6). Therefore, *SlGSNOR1* silencing may trigger a putative mechanism that results in *S*-nitrosylation of NADPH oxidase, which compromised thermotolerance. Actually, NADPH oxidase has been shown to be directly inhibited by *S*-nitrosylation during pathogen defense in Arabidopsis (Yun et al., 2011). Although detailed mechanism for inhibition of apoplastic H_2_O_2_ production by silencing of *SlGSNOR1* has not been revealed in this study, it is clear that H_2_O_2_ treatment recovered the thermotolerance of *SlGSNOR1*-silenced plants (Figure 7). This highlights the importance of maintaining appropriate level of ROS for adaptation to high temperature. More importantly, it added complexity to the crosstalk between ROS and NO in response to high temperature. NO is essential and acts downstream of RBOH-dependent ROS for heat shock response in Arabidopsis (Wang et al., 2014). However, SNO accumulation due to *SlGSNOR1* silencing inhibited early production of ROS in response to high temperature in this study. In addition, suppression of apoplastic H_2_O_2_ production through *SlRBOH1* silencing inhibited the induction of GSNOR activity, leading to SNO accumulation at high temperature (Figure 6). In this sense, ROS and SNO antagonistically regulate the accumulation of each other. Previously, a balance model for NO and ROS interactions was proposed, where the presence of both NO and ROS is critical for defense response, whereas direct chemical reaction between NO and ROS attenuated defense response (Delledonne et al., 2001). Similarly, a higher NO level as a result of GSNOR mutation counteracts the paraquat-mediated superoxide, resulting in reduced cell death in Arabidopsis (Chen et al., 2009). In this study, it appears that GSNOR is important for maintaining a balanced NO/ROS interaction in response to high temperature. When excessive SNO accumulates in *SlGSNOR1*-silenced plants at high temperature, the accumulation of apoplastic H_2_O_2_ was inhibited, leading to attenuated heat stress response. Environmental stress triggers a ROS wave which harbors stimulus-specific information for initiation of an appropriate response (Mittler et al., 2011; Xia et al., 2015). Considering the NO/ROS interaction, we hypothesize that the level of NO relative to that of ROS is actively regulated through robust downregulation or upregulation of GSNOR in plant stress response. This may explain why response of GSNOR varied, depending on stress types and plant species (Corpas et al., 2008; Chaki et al., 2011; Airaki et al., 2012).

**FIGURE 9:**
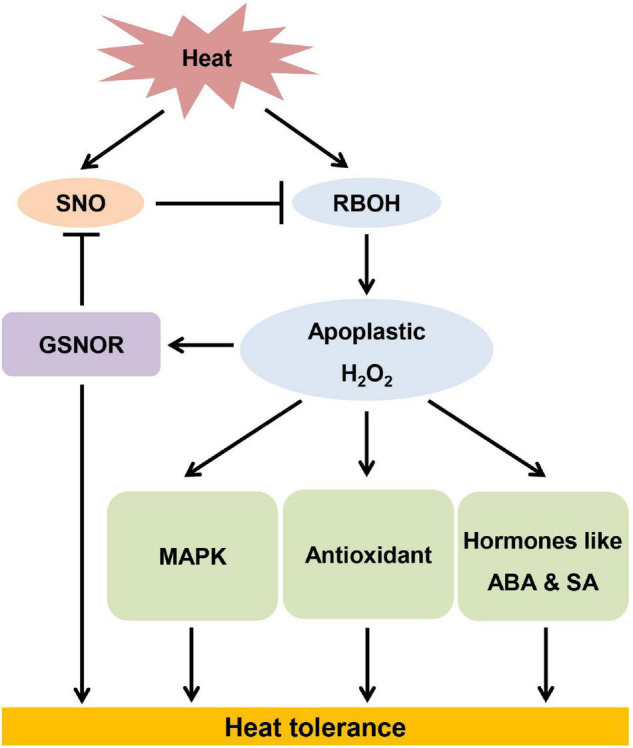
The model of *S*-nitrosoglutathione reductase contributing to thermotolerance by modulating high temperature-induced apoplastic H_2_O_2_.

In summary, GSNOR contributes to thermotolerance through regulation of RBOH-dependent apoplastic H_2_O_2_ (Figure 9). Heat stress induces accumulation of SNO, whose level is controlled by GSNOR. Excessive accumulation of SNO due to loss of function of *GSNOR* leads to inhibition of RBOH activity. Conversely, RBOH-mediated apoplastic H_2_O_2_ is essential for activation of GSNOR, which prevent nitrasative stress. On the other hand, apoplastic H_2_O_2_ activates MAPK, antioxidants and stress hormones, which are all involved in thermotolerance.

## Data Availability Statement

The original contributions presented in the study are included in the article/Supplementary Material, further inquiries can be directed to the corresponding author/s.

## Author Contributions

X-JX and J-QY conceived, designed, and supervised the experiments. XS, TW, and YZ conducted the experiments and analyzed the data. XS and X-JX prepared the first draft of the manuscript. XS and TW contributed to final editing of the manuscript. All authors contributed to the article and approved the submitted version.

## Conflict of Interest

The authors declare that the research was conducted in the absence of any commercial or financial relationships that could be construed as a potential conflict of interest.

## Publisher’s Note

All claims expressed in this article are solely those of the authors and do not necessarily represent those of their affiliated organizations, or those of the publisher, the editors and the reviewers. Any product that may be evaluated in this article, or claim that may be made by its manufacturer, is not guaranteed or endorsed by the publisher.
