# Supplementary material for: S-Nitrosoglutathione Reductase Contributes to Thermotolerance by Modulating High Temperature-Induced Apoplastic H2O2 in Solanum lycopersicum
Source: Front Plant Sci. 2022 Apr 12;13:862649. doi: 10.3389/fpls.2022.862649 (PMC9042256; doi:10.3389/fpls.2022.862649)
Supplement: Supplementary file 2 [file Table_1.DOCX]

**Supplemental figure 1. Negative control for H_2_O_2_ detection using ascorbate.**

**
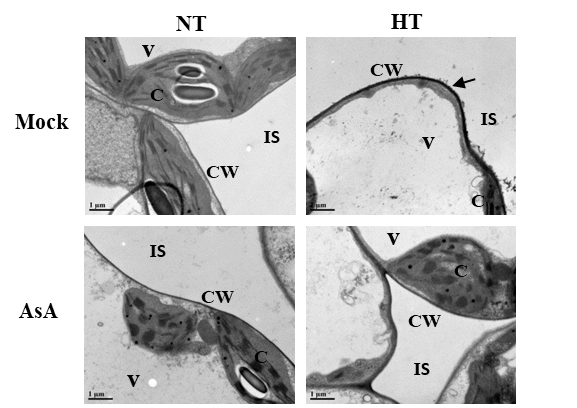
**

**Subcellular localization of H_2_O_2_ leaf cells using CeCl_3_. Black arrows indicate apoplastic H_2_O_2_ accumulation. Samples were taken at 3 h after heat stress. C, chloroplast; CW, cell wall; IS, intercellular space; V, vacuole. Data are means of five replicates (±SD). Ascorbate (AsA) was used as negative control. NT, normal temperature; HT, high temperature.**
