# Supplementary material for: S-Nitrosoglutathione Reductase Contributes to Thermotolerance by Modulating High Temperature-Induced Apoplastic H2O2 in Solanum lycopersicum
Source: Front Plant Sci. 2022 Apr 12;13:862649. doi: 10.3389/fpls.2022.862649 (PMC9042256; doi:10.3389/fpls.2022.862649)
Supplement: Supplementary file 3 [file Table_2.DOCX]

| **Gene name** | **Functional annotations** | **Primer sequences (5’-3’)** | **Accession numbers** |
| --- | --- | --- | --- |
| *RBOH1* | Respiratory Burst Oxidase Homolog1 | F: CATTTGATTTGGGACA; R: CTTCAACAAACTCCTCC | Solyc08g081690.2.1 |
| *GSNOR1* | *S*-nitrosogutathione reductase | F: GTCGTGTTTGGAAGGGAACT; R: ATGTCCGCAAGTGTCATGTT | Solyc09g064370.2.1 |
| *Cu/Zn-SOD* | Cu/Zn-superoxide dismutase | F: CTCCTGGAGATGAAATCCGT; R: AAGTGCTCGTCCAACAACTG | Solyc11g066390.1.1 |
| *cAPX* | Cytosolic ascorbate peroxidase | F: GAGGTGGAGCTAATGGAAGC; R: ACTGGCCAGCTGGAATAAAT | Solyc06g060260.2.1 |
| *GR* | Glutathione reductase | F: TTGGTGGAACGTGTGTTCTT; R: TCTCATTCACTTCCCATCCA | Solyc09g091840.2.1 |
| *CAT* | Catalase | F: TGATCGCGAGAAGATACCTG; R: CTTCCACGTTCATGGACAAC | Solyc12g094620.1.1 |
| *HSP90* | Heat shock protein 90 | F: GTCCAGCAAGAAGACGATG; R: GCAGCAAAGGTATTAGGGT | Solyc03g007890.2.1 |
| *Actin* | Actin | F: TGGTCGGAATGGGACAGAAG; R: CTCAGTCAGGAGAACAGGGT | Solyc03g078400.2.1 |

**Supplemental table 1. Primers used in this study for gene expression analysis by qRT-PCR**
